# Acknowledgement to Reviewers of *Vaccines* in 2014

**DOI:** 10.3390/vaccines3010020

**Published:** 2015-01-08

**Authors:** Vaccines Editorial Office

**Affiliations:** MDPI AG, Klybeckstrasse 64, CH-4057 Basel, Switzerland

The editors of *Vaccines* would like to express their sincere gratitude to the following reviewers for assessing manuscripts in 2014:

Abel, Kristina
Abou-El-Enein, Mohamed
Acedo, L.
Afonso, Nelia
Agadjanyan, Michael
Arens, Ramon
Atif, S. M.
Ato, Manabu
Behrens, Sven-Erik
Beverley, P. C. L.
Buddle, Bryce
Calvo-Pinilla, Eva
Chackerian, B.
Chambers, Thomas M.
Chevalier, Christophe
Chong, Pele
Cox, Josephine H.
Daftarian, Pirouz
Dahlström, Lisen Arnheim
Dalton, John P.
Dennehy, Kevin M.
Dexheimer, Judith W.
Dow, Steven
Estepa, Amparo
Fast, Patricia
Felber, Barbara K.
Feodorova, Valentina A.
Fomsgaard, Anders
Forde, Jonathan
Fu, Linda Y.
Fujita, Yoshio
Ghochikyan, Anahit
Gianella, Sara
Girard, Marc P.
Griffiths, Kristin
Guzman-Cottrill, Judith A.
Haks, M. C.
Hanke, Tomáš
Hellstrom, Karl Erik
Hemmatzadeh, Farhid
Hinrichs, Wouter L. J.
Hofstetter, Annika M.
Houen, Gunnar
Hu, Linden
Hurwitz, J. L.
Iafusco, Dario
Khurana, Surender
Klasse, Per-Johan
Klug, Bettina
Kobinger, Gary
Koblin, Beryl
Krammer, Florian
Kunz, Horst
Lambracht-Washington, Doris
Lapatra, Scott
Laurie, Karen
Lavelle, Ed
Leclerc, Denis
Lee, Shee Eun
Lomonossoff, George P.
Lonez, Caroline
Macphail, Catherine
Maier, Bernhard
Makidon, Paul
Maltezou, Helen C.
Marillonnet, Sylvestre
Marrack, Philippa
McGettigan, James P.
Medina, Marcela
Mehrad, Borna
Meseda, Clement
Morris, John C.
Motin, Vladimir L.
Nagata, Toshi
Nandakumar, Kutty Selva
Nayak, Jennifer L.
Nieva, José L.
Niidome, Takuro
Niller, Hans-Helmut
Palese, Peter
Palmu, Arto A.
Pannaraj, Pia S.
Paran, Nir
Perrett, Kirsten P.
Petrovsky, Nikolai
Piganelli, Jon
Pistello, Mauro
Raghavan, Sukanya
Rebchook, Greg
Robinson, Harriet
Rockman, Steven
Rosenmann, Hanna
Salgado, Cassandra D.
Salio, Mariolina
Salvador, Aiala
Santibanez, Tammy A.
Sharma, Sherven
Shimada, Masaru
Siccardi, Antonio G.
Siddiqui, Afzal A.
Skwarczynski, Mariusz
Slack, Mary
Smerdou, Christian
Strobl, Herbert
Sui, Yongjun
Suryadevara, Manika
Tan, Tina Q.
Tartour, Eric
Tregoning, John
Tripp, Daniel W.
Ueberla, Klaus
Ueda, Yutaka
Van Wagoner, Nicholas J.
Vanrompay, Daisy
Wang, Xin
Werling, Dirk
White, Laura
Wu, Mei X.
Xing, Zhou
Yamanaka, T.
Yano, Akira
Yasutomi, Yasuhiro
Yilma, Tilahun D.
Zaman, Mehfuz
Zhang, Guoquan

